# Targeted Fisetin-Encapsulated β-Cyclodextrin Nanosponges for Breast Cancer

**DOI:** 10.3390/pharmaceutics15051480

**Published:** 2023-05-12

**Authors:** Alaa R. Aboushanab, Riham M. El-Moslemany, Amal H. El-Kamel, Radwa A. Mehanna, Basant A. Bakr, Asmaa A. Ashour

**Affiliations:** 1 Department of Pharmaceutics, Faculty of Pharmacy, Alexandria University, Alexandria 21525, Egypt; 2 Department of Medical Physiology, Faculty of Medicine, Alexandria University, Alexandria 21525, Egypt; 3 Center of Excellence for Research in Regenerative Medicine and Applications (CERRMA), Faculty of Medicine, Alexandria University, Alexandria 21525, Egypt; 4 Department of Zoology, Faculty of Science, Alexandria University, Alexandria 21525, Egypt

**Keywords:** phytomedicine, nanosponges, lactoferrin, bioavailability, MDA-MB-231 cells, caspase-3, cyclin-D1

## Abstract

Fisetin (FS) is considered a safer phytomedicine alternative to conventional chemotherapeutics for breast cancer treatment. Despite its surpassing therapeutic potential, its clinical utility is hampered by its low systemic bioavailability. Accordingly, as far as we are aware, this is the first study to develop lactoferrin-coated FS-loaded β-cyclodextrin nanosponges (LF-FS-NS) for targeted FS delivery to breast cancer. NS formation through cross-linking of β-cyclodextrin by diphenyl carbonate was confirmed by FTIR and XRD. The selected LF-FS-NS showed good colloidal properties (size 52.7 ± 7.2 nm, PDI < 0.3, and ζ-potential 24 mV), high loading efficiency (96 ± 0.3%), and sustained drug release of 26 % after 24 h. Morphological examination using SEM revealed the mesoporous spherical structure of the prepared nanosponges with a pore diameter of ~30 nm, which was further confirmed by surface area measurement. Additionally, LF-FS-NS enhanced FS oral and IP bioavailability (2.5- and 3.2-fold, respectively) compared to FS suspension in rats. Antitumor efficacy evaluation in vitro on MDA-MB-231 cells and in vivo on an Ehrlich ascites mouse model demonstrated significantly higher activity and targetability of LF-FS-NS (30 mg/kg) compared to the free drug and uncoated formulation. Consequently, LF-FS-NS could be addressed as a promising formulation for the effective management of breast cancer.

## 1. Introduction

Nanosponges (NS) are promising polymeric colloidal systems. As the name implies, NS are nano-sized porous structures offering ideal properties for drug delivery. NS can be synthesized from various polymers and copolymers, such as hyper cross-linked polystyrenes [1], ethyl cellulose [2], or cyclodextrins [3]. Cyclodextrins (CDs) are generally recognized as safe (GRAS), as listed by the Food and Drug Administration (FDA) [4]. Including β-CD as the most studied and most frequently used, CDs are cyclic oligosaccharides with a cage-like structure and a distinct cone-shaped lipophilic cavity [5]. This unique structure contributes to their ability to form inclusion complexes with various molecules, resulting in enhanced aqueous solubility and protection against degradation [6]. However, the ease of dissociation of the formed complexes upon dilution, in addition to the inability to complex certain molecules, are considered major drawbacks [6]. To overcome these limitations, structural modifications of CDs have been suggested to increase the inclusion capacity and allow for a wider scope of biological applications [7]. Of these, cyclodextrin-based nanosponges (CDNS) have recently attracted attention and been synthesized by polymer cross-linking to form a highly porous branched matrix [3]. The obtained amphiphilic spongy structure confers the ability to accommodate hydrophobic molecules in the CD cavities and fewer lipophilic molecules in the more hydrophilic outer polymeric network with high loading capacity and controlled drug release [8]. Furthermore, CDNS offer other advantages, such as being highly biocompatible, biodegradable, and of low cytotoxicity [9]. In addition, this nanosystem was shown to improve permeation across biological barriers and enhance the bioavailability of active substances [6]. As a controlled drug delivery system, CDNS has been explored for oral, topical, and parenteral drug delivery for numerous applications, such as anticancer, antiviral, antihypertensive and antiplatelet therapy [5,10]. One of the most relevant fields is drug delivery for cancer therapy [9,11], including breast cancer. In this aspect, CDNS showed improved anticancer drug effects both in vitro [12,13] and in vivo [14].

The breast cancer (BC) burden has been rising sharply over the past decades. Having replaced lung cancer, it is now the most diagnosed cancer worldwide, representing a quarter of all cancer cases in females. Despite the significant advances in BC treatment, a continuous rapid increase in the number of new cases and deaths is recorded, with a 40% projection expected especially in low human developing index countries by 2040 compared to 2020 [15]. This could be imputed to the limitations of chemotherapy, including non-selectivity to cancer cells, multidrug resistance (MDR), and ineffective inhibition of tumor growth, metastasis, and recurrence. Thus, recent strategies for the prevention and treatment of BC focused on the use of herbal medicine as a safe, effective, and low-cost alternative to cytotoxic drugs. Considerable anticancer activity in natural phytochemicals has been confirmed against different cancers via numerous in vitro and in vivo studies [16]. Flavonoids are plant polyphenols exhibiting various beneficial properties for breast cancer therapy [17]. They were shown to affect growth and proliferation via cell cycle arrest, necrosis, and apoptosis. Furthermore, they exhibit antioxidant, anti-inflammatory, and anti-mutagenic properties [18]. A potent flavonoid found in various fruits and vegetables and exhibiting cytotoxic activity is fisetin (3,3′,4′,7-tetrahydroxy flavone, FS) [19]. The anticancer effect of FS on different breast cancer cell lines including triple-negative breast cancer (TNBC) cells was manifested [20,21]. Among several mechanistic studies on different breast cancer cell lines, FS was shown to inhibit cell proliferation and metastasis, prevent cell cycle progression, induce apoptosis, cause cleavage of poly ADP ribose polymerase (PARP), and modulate Bcl-2 family protein expression [22]. Moreover, FS was capable of suppressing PKCα/ROS/ERK1/2 and p38 MAPK signaling pathway activation, reducing NF-κB activation, and lowering TET1 expression in a concentration- and time-dependent manner [22,23]. It also reverses the epithelial to mesenchymal transition (EMT) process mediated by the PTEN/Akt/GSK-3β signaling pathway [20].

Despite its bioactive potential in the prevention and treatment of different cancer conditions, the clinical applications of FS have been impeded by its highly lipophilic nature, and, hence, limited aqueous solubility [24]. Furthermore, it is rapidly metabolized, enzymatically degraded, and liable to p-glycoprotein efflux following oral administration, which leads to a short half-life and poor bioavailability [24,25]. These curtailments interfere with FS bioaccessibility, necessitating the development of novel oral drug delivery approaches, such as loading into nanosystems [26,27]. Furthermore, the nanoparticles’ surface could be modified with active ligands, such as folic acid, hyaluronic acid, chondroitin sulfate, and lactoferrin, for selective and efficient accumulation at tumor sites via targeting specific receptors overexpressed on BC cells [26]. Lactoferrin (LF) is an iron-binding glycoprotein possessing a strong affinity to transferrin receptors overexpressed on breast cancer cells [28]. It is capable of either promoting or inhibiting cell proliferation and migration depending on whether the cell it acts upon is normal or cancerous, respectively [29]. Moreover, being a part of the innate immune system, LF boosts adaptive immune response, thus, preventing or inhibiting cancer development [29].

In this regard, the current study aimed to develop FS-loaded cyclodextrin nanosponges (FS-NS) coated with the bioactive protein LF to enhance FS bioavailability and anticancer activity. This novel nanosystem would provide multiple advantages, including high drug loading and entrapment efficiency with sustainment of release and an improvement in the anticancer activity of FS via both passive targeting by enhanced permeability and retention (EPR) effect and active targeting by the LF coating. Following formulation optimization and in vitro characterization, the change in FS pharmacokinetic parameters upon loading into proposed LF-FS-NS relative to FS suspension and uncoated FS-NS was assessed. Moreover, the cytotoxicity and cellular uptake of the test formulations were assessed on MDA-MB-231, a human TNBC cell line. A murine Ehrlich ascites breast cancer mouse model was used to assess in vivo anticancer efficacy.

## 2. Materials and Methods

### 2.1. Materials

β-cyclodextrin, diphenyl carbonate (DPC), coumarin 6 (C6), and quercetin were purchased from Sigma-Aldrich (St. Louis, MO, USA). Lactoferrin (LF) was obtained from Lactoferrin.co (Frankfurt, Germany). Fisetin (FS) was from Arctom Scientific (Agoura Hills, CA, USA). HPLC-grade acetonitrile, methanol, dimethylformamide (DMF), and formic acid were purchased from Fischer Scientific (Loughborough, UK). Hoechst 33342 stain, an annexin V FITC/propidium iodide (PI) kit, fetal bovine serum (FBS), and Dulbecco’s modified Eagle’s medium (DMEM) were purchased from Sigma-Aldrich (St. Louis, MO, USA). Penicillin and streptomycin solution (100 U/mL each) were from BioWhittaker^®^ (Lonza, Belgium). 3-[4, 5-dimethylthiazol-2-yl]-2,5-diphenyl tetrazolium bromide (MTT) was purchased from Serva (Heidelberg, Germany). PCR primers were obtained from Eurofins Scientific (Luxembourg). A one-step RT qPCR kit (SYBR Green with low ROX) was from Enzynomics Co. Ltd., Yuseonggu, Daejeon, Korea. All the other reagents were of analytical grade and were used without further purification.

### 2.2. Preparation of Blank Nanosponges

NS formulations were prepared using diphenyl carbonate (DPC) cross-linker as previously described, with some modifications [30]. In brief, β-cyclodextrin (CD) was dissolved in N, N-dimethylformamide (DMF), and DPC was added in a molar ratio of 1:6. The reaction mixture was mixed under magnetic stirring at 450 rpm for 20 min in a water bath at 80 °C. For optimization, the effects of heating the reaction mixture at 90, 120, or 150 °C for different time intervals (2 or 5 h) while using different volumes of DMF (3 or 6 mL) were investigated. The product was subjected to 5 cycles of washing with deionized water and acetone and then left to dry in a desiccator for 48 h. The purified powdered formulation was ground in a mortar and accurately weighed to calculate the percentage yield using the following equation:

(1) $$\%\;Yield=\frac{Weight\;of\;the\;nanosponge}{Weight\;of\;\beta -CD+Weight\;of\;DPC}\times 100$$

The obtained powder (14 mg) was dispersed in 2 mL deionized water and sonicated using a probe sonicator (Bandelin Sonoplus, Germany) at 60% amplitude for 10 min. Homogenization at 10,000 rpm for 10 min was subsequently carried out to obtain a nano-dispersion (NS).

### 2.3. Ferric Chloride Test

To qualitatively verify NS formation, the FeCl_3_ test was performed to detect the presence of phenols formed as a by-product during the cross-linking esterification reaction between β-CD and DPC. Unwashed NS (10 mg) were dispersed in 3 mL of deionized water followed by the addition of 1 mL FeCl_3_ solution. The appearance of a deep violet color indicated the presence of phenol, proving NS formation [30].

### 2.4. Preparation of Fisetin-Loaded Nanosponges

For the preparation of fisetin-loaded nanosponges (FS-NS), the drug was dissolved in ethanol and added to the NS dispersion in a ratio of 1:4 to obtain a final drug concentration of 1.75 mg/mL. For complete drug loading, the mixture was subjected to 5 min sonication in a bath sonicator followed by overnight stirring [10].

### 2.5. Preparation of Lactoferrin (LF)-Coated FS-NS

FS-NS formulation dispersion (2 mL) was dropped into 100 µL of LF solution in PBS pH 6.5 and stirred at 500 rpm for 30 min. Different LF concentrations (25–100 mg/mL) were tested. Efficient LF coating was verified by size and ζ-potential measurements.

### 2.6. Physicochemical Characterization

#### 2.6.1. Surface Area and Porosity Analysis

Specific surface area and porosity of the prepared NS were measured using a nitrogen absorption–desorption isotherm (Belsorp-Mini II analyzer, Japan). The sample (0.2 g) was degassed for 3 h before analysis. The surface area and porosity were determined using Brunauer–Emmett–Teller (BET) and Barrett–Joiner–Halenda analyses [31].

#### 2.6.2. Microscopical Examination

##### Scanning Electron Microscopy (SEM)

The surface morphology and porosity of blank NS were evaluated using a scanning electron microscope (SM-IT200; JEOL, Tokyo, Japan). The NS dispersion was mounted on a metal stub, air-dried, and sputter-coated with gold before the examination.

##### Transmission Electron Microscopy (TEM)

Transmission electron microscopy (TEM) (model JEM-100CX, JEOL, Japan) was used to further investigate the morphology as well as to determine the average size of NS, FS-NS, and LF-FS-NS [32]. Dispersions were dropped on carbon-coated copper grids, stained with uranyl acetate (1% *w*/*v*), and air-dried before the examination. For the determination of particle size (PS), 50 measurements from different fields were carried out using image-analysis software [31] (Fiji 1.52p; National Institutes of Health, Bethesda, MD, USA) and the polydispersity index (PDI), calculated by the following equation:

(2) $$PDI={\left(\frac{SD}{d}\right)}^{2}$$

where *SD* is the standard deviation and *d* is the average diameter.

#### 2.6.3. ξ-Potential Measurement

ζ-potential of different formulations (NS, FS-NS, LF-FS-NS) was determined using a Malvern Zetasizer (Nano-ZS Series DTS 1060, Malvern Instruments, UK). Measurements were performed at a fixed angle of 173° at 25 °C. Formulations were adequately diluted with deionized distilled water in the ratio 1:100 and measured in triplicate.

#### 2.6.4. Entrapment Efficiency and Drug Loading Determination

Fisetin entrapment efficiency% (EE%) was carried out using the dialysis technique [33]. FS-NS and LF-FS-NS dispersions (0.5 mL) were placed in a dialysis bag (Visking 36/32, 28 mm, MWCO 12–14 KDa; Serva, Heidelberg, Germany) and then immersed in 26 mL PBS (pH 7.4) containing 0.1% Tween^®^ 80 to maintain sink conditions, before being centrifuged at 25 °C and 500 rpm for 30 min using a high-speed cooling centrifuge (Model 3K-30; Sigma Laborzentrifugen GmbH, Osterode, Germany). The free unentrapped drug in the eluent was quantified spectrophotometrically at 360 nm using a UV–visible spectrophotometer (Cary 60 UV–visible spectrophotometer, Agilent, Santa Clara, CA, USA). Linearity was checked in the range 5–15 µg/mL with a coefficient of determination R^2^ = 0.99. For % drug loading (DL%) determination, NS was redispersed in ethanol following centrifugation. The amount of FS analyzed denoted the weight of FS in NS formulations [10]. EE% and DL% were calculated using the following equations:

(3) $$EE\;\%=\frac{Total\;drug\;amount\;-\;unentraped\;drug\;amount}{Total\;drug\;amount}\times 100$$

(4) $$DL\;\%=\frac{Drug\;amount\;in\;NS}{Total\;weight\;of\;NS}\;\times \;100$$

#### 2.6.5. In Vitro Drug Release

In vitro release of FS from both FS-NS and LF-FS-NS in comparison to the drug solution was carried out using the dialysis bag method. The drug solution was prepared in an aqueous PEG 400 solution (50% *v*/*v*). NS dispersions (0.5 mL equivalent to 0.9 mg FS) were added into dialysis bags and then placed into 30 mL PBS (pH 7.4) with 0.1% *w*/*v* Tween^®^ 80, maintaining sink conditions. The experiment was carried out in a thermostatically controlled shaking water bath at 37 °C and 100 rpm. At different time intervals (1–24 h), samples (1 mL) were withdrawn and replaced with fresh medium. The drug concentration was determined spectrophotometrically at 360 nm. Then, the percentage of cumulative FS released was calculated in triplicate.

Drug release kinetics from NS dispersions were assessed using model-dependent methods [34] calculated by the Excel add-in DDsolver [35].

#### 2.6.6. Fourier Transform Infrared Spectroscopy (FTIR)

Prior to analysis, FS-NS dispersion was dried using Aerosil^®^ 200, as previously described [36]. Briefly, FS-NS aqueous dispersion was mixed with Aerosil^®^ 200 in a ratio of 4:1, and then the mixture was allowed to dry overnight in a desiccator. The FTIR spectra of Aerosil^®^ 200, β-CD, DPC, NS, FS, and FS-NS were obtained using an FTIR spectrometer (PerkinElmer Inc., Waltham, MA, USA). Tested samples were mixed with KBr (1:100 *w*/*w*) and compressed to form a disc that was scanned in the range 4000–500 cm^−1^.

#### 2.6.7. X-ray Powder Diffractometry (XRD)

The crystallinity of Aerosil^®^ 200, NS, FS, and FS-NS was assessed using XRD (XRD-7000 X-ray diffractometer, Bruker D2-Phaser; Madison, WI, USA). The diffraction pattern was performed in a step scan model of 30 kV and 30 mA with a scanning region for the diffraction angle, 2θ, from 0 to 100°, with step size 0.02°. 

### 2.7. In Vitro Cell Culture Studies

In vitro cell culture studies were performed on the human breast cancer cell line (MDA-MB-231) obtained from ATCC (HTB-26™) and cultured at CERRMA (Center of Excellence for Research in Regenerative Medicine and its Applications), Faculty of Medicine, Alexandria University. Cells were grown in Dulbecco’s modified Eagle’s medium (DMEM)-high glucose, enriched with (10 % *v*/*v*) fetal bovine serum (FBS) and antibiotics (100 U/mL penicillin, 100 μg/mL streptomycin). Cells were maintained in a 5% CO_2_ incubator at 37 °C. 

#### 2.7.1. MTT Cytotoxicity Assay

MDA-MB-231 cells were grown as monolayer cultures in the culture media mentioned above and monitored daily using the phase-contrast inverted microscope (CKX41SF; Olympus) for their growth and morphology. Media were changed every 2–3 days. When confluent, cells were plated into 96-well plates with a uniform seeding density of 5 × 10^3^ cells/well and allowed to adhere for 24 h. The medium was then replaced with medium containing different concentrations of FS, FS-NS, and LF-FS-NS ranging from 10 to 100 µg/mL and the similarly diluted blank formulations NS and LF-NS, and then incubated for 48 h at 37  °C and 5% CO_2_. The media were then aspirated, replaced by MTT solution, and incubated for another 4 h. Finally, the formazan blue crystals were dissolved in DMSO, and the absorbance was measured at 570 nm by an ELISA well-plate reader (Tecan, Infinite F50, Männedorf, Switzerland). The values obtained were compared with the control, which was regarded as 100% living cells. Experiments were performed in triplicate [26].

#### 2.7.2. Apoptosis Assay (Annexin V-FTIC/Propidium Iodide Assay)

Annexin V assay was used to assess the apoptotic effect of FS, FS-NS, and LF-FS-NS [26]. Cells were incubated in 6-well plates in a density of 2 × 10^5^ cells/well and left to adhere for 24 h at 37 °C and 5% CO_2_. Cells were treated with samples equivalent to half the IC_50_ value calculated from the cytotoxicity experiment for 24 h. Cells were then trypsinized, collected by centrifugation at 2000 rpm, and stained with annexin V-fluorescein isothiocyanate (FITC) (50 µg/mL, 5 µL) and propidium iodide (PI) (50 µg/mL, 5 µL) as per the manufacturer’s protocol. Analysis of apoptotic cells was performed by 20,000 cells gating by flow cytometry (BD FACSCalibur™ flow cytometer, San Jose, CA, USA). The experiment was performed in triplicate with representative images provided.

#### 2.7.3. Cell Migration by Wound Healing Assay

The effect of FS, FS-NS, and LF-FS-NS on the migration ability of MDA-MB-231 cells was examined by the wound healing assay [37]. Cells were seeded in 12-well plates and incubated at 37 °C with 5% CO_2_ until confluent. A 100 µL pipette tip was used to make a single scratch in the cell monolayer, and an image of the scratch was captured using the phase-contrast inverted microscope (CKX41SF; Olympus). The cells were then treated with concentrations corresponding to half IC_50_ values and incubated for 24 h. Following incubation, the scratch width was imaged. Images were analyzed using NIH ImageJ software, and the ratio of cell migration was calculated as the percentage of the remaining cell-free area in comparison to the area of the initial scratch.

#### 2.7.4. Cellular Uptake

Fluorescently labeled NS and LF-NS were prepared using coumarin 6 (C6). A similar loading procedure was adopted with the addition of the dye to ethanol instead of FS. Briefly, 100 μL of freshly prepared C6 solution (250 μg/mL in ethanol) was added to 2 mL of NS and stirred overnight. MDA-MB-231 cells were seeded in 6-well plates at an initial density of 8  ×  10^4^ cells/well on coverslips. Then, 24 h later, C6-labeled NS formulations, both uncoated and LF-coated, and the free dye were added to the corresponding wells and incubated at 37  °C and 5% CO_2_ for 4 h. The cells were then washed three times with PBS and fixed with 4% paraformaldehyde at room temperature for 30 min in the dark. Cell nuclei were stained with Hoechst 33,342 for another 20 min. The cells were observed using laser scanning confocal microscopy (LEICA, DMi8, Mannheim/Wetzlar, Germany), and the fluorescent signals of the conjugated formulations were analyzed and compared with the control and free C6. Image processing was conducted using the Leica Application Suite X (LAS X) software. The uptake study was conducted in triplicates. Finally, quantification of fluorescence intensity in the obtained images was performed using ImageJ software, National Institute of Health, NIH (version 1.45s) [38].

### 2.8. In Vivo Studies

#### 2.8.1. Pharmacokinetic Study

##### Study Design

The study was conducted on female Wistar rats (200 ± 20 g) that were kept under standard conditions of light, temperature, and humidity, with free access to food and water during the study.

Rats were randomly divided into 6 groups (each of 6 animals). Animals were fasted overnight prior to the experiment. A single dose of FS suspension, FS-NS, and LF-FS-NS equivalent to 30 mg FS/kg was administered either orally by gastric gavage or by IP injection. FS suspension was prepared in 0.5% *w*/*v* carboxymethyl cellulose sodium for oral administration and in 10% PEG 400 saline solution for IP injection. Blood samples were collected via the orbital plexus under anesthesia at predetermined time points (0.25, 0.5, 1, 3, 5, 7, and 24 h) in EDTA-containing tubes. Blood samples were centrifuged at 4000 rpm for 10 min. Plasma samples were frozen and maintained at −80 °C pending analysis.

##### Quantification of FS in Plasma Samples 

FS in plasma samples was analyzed using a reported HPLC-UV method for the quantification of FS in plasma samples, with slight modification [39]. Plasma samples (300 µL) were vortex mixed with 40 μL quercetin (1 μg/mL) as an internal standard and an equal volume of methanol acidified with 0.5% *v*/*v* formic acid for plasma protein precipitation for 1 min, and then centrifuged at 10,000 rpm for 10 min. The obtained supernatants were filtered through 0.22 μm syringe PTFE filters. The HPLC instrument (Agilent 1260 infinity, Germany) equipped with a quaternary pump (G1311C) and a UV variable wavelength detector (G1314F) was used. Samples (100 μL) were manually injected in a reversed-phase C_18_ column (Agilent HC-C_18_(2), 4.6 × 150 mm, 5 μm). The mobile phase was a mixture of acetonitrile and water acidified with 0.5% *v*/*v* formic acid in the ratio of 40:60. The flow rate was maintained at 1 mL/min, and detection was performed at 360 nm. Quantification was achieved using a calibration curve for peak area ratios of FS/quercetin in spiked plasma obtained under the same conditions.

##### Pharmacokinetic Data Analysis

The plasma concentrations vs. time data were analyzed by a non-compartmental pharmacokinetic model using the Excel pharmacokinetic solver add-in [40], and pharmacokinetic parameters were calculated. Results were expressed as mean ± standard deviation (SD) (n = 6). 

#### 2.8.2. Antitumor Efficacy Evaluation

##### Experimental Design

Efficacy and toxicity studies were performed on female Swiss albino mice (7–8 weeks of age, about 20–25 g). Animals were maintained under controlled temperature and humidity with a 12 h dark/light cycle, with free access to food and water. 

Mammary tumors were induced in mice using the Ehrlich ascites tumor (EAT) model [26]. EAT cells, obtained from the National Cancer Institute, Cairo, Egypt, were properly diluted (~2 × 10^6^) and subcutaneously injected into the left mammary fat pad of mice. The tumor volume was evaluated until it reached ~100 mm^3^ (12 days post-inoculation). Tumor-bearing animals were then randomly assigned into four groups (n = 6) as follows: positive control group (untreated), FS suspension in 10% PEG 400 saline solution, FS-NS, and LF-FS-NS administered by IP injection. Treated groups received 7 doses on an alternate-day dosing regimen of either FS suspension or NS formulations equivalent to 30 mg FS/kg. A negative control group was included for comparison. At the end of the study, mice were sacrificed, and tumors were excised and washed with ice-cold phosphate buffer. Tumor weight was determined, and then tumors were divided into portions for further assessment.

##### Assessment of Tumor Growth

The percentage change in tumor volume compared to baseline volume was determined as an indicator of tumor growth. Tumor volume was assessed twice weekly. A vernier caliper was used to measure the tumor length (major axis) and width (minor axis), and then tumor volume was calculated according to the following equation [26]:

(5) $$Tumor\;volume=length\;\times \;{\left(width\right)}^{2}\;\times \;0.5$$

##### Tumor Biomarkers

Excised tumors were homogenized in phosphate buffer saline (pH 7.4) to make a final 10% tissue homogenate that was used for the quantitative determination of tumor growth biomarkers. ELISA kits were used to assess cyclin-D1 level (Mouse cyclin-D1 ELISA Kit, EIAal™, Waltham, MA, USA) as an indicator of proliferation rate and the anti-apoptotic BCl-2 protein (catalog no. CSB-E08855m). Quantification was performed according to the manufacturer’s protocol.

The relative expression of Bax and caspase-3 genes in tumor tissues, using quantitative real-time reverse transcriptase polymerase chain reactions (qRT-PCR), was performed. Forward and reverse primer sequences for PCR amplification are shown in Table 1. The RT-PCR reactions were run in triplicate with signal collection at the end of each cycle. An internal housekeeping gene (GAPDH) was applied to normalize relative transcript levels. The comparative threshold cycle (ΔΔCt) method was used to determine sample differences. 

##### Histopathological Examination

Excised tumor specimens were fixed in 10% formalin for 24 h. Sections (5 μm thickness) were cut, stained with hematoxylin and eosin (H&E) stain for 5 min, dehydrated in alcohol, and mounted in Canada balsam prior to microscopical examination. 

#### 2.8.3. In Vivo Toxicity Study

The effect of NS formulations vs. FS suspension on general animal health during the treatment course was evaluated. Change in body weight was recorded on a weekly basis. Following sacrifice, the liver, kidney, and spleen were excised and fixed in 10% formalin for histopathological examination. Furthermore, liver and kidney functions were evaluated by measuring serum alanine aminotransferase (ALT), aspartate aminotransferase (AST), urea, and creatinine in comparison to healthy animals. 

### 2.9. Statistical Analysis

All experiments were conducted in triplicate, and results were represented as mean ± SD. Statistical analyses were performed using an unpaired Student’s *t*-test and one-way analysis of variance (ANOVA) followed by a post-hoc Tukey’s test for multiple comparisons using GraphPad Prism (Version 7.04, San Diego, CA, USA). The level of significance was set at *p* ≤ 0.05. 

## 3. Results and Discussion

### 3.1. Preparation and Optimization of β-CD-NS

NS formulations were prepared using DPC as a cross-linker for β-CD hydroxyl groups with the formation of new carbonate bonds [30]. A molar ratio 1:6 of β-CD:DPC was selected based on previous reports showing a higher percentage yield compared to other ratios tested [10,30]. Optimization of the cross-linking reaction conditions was carried out by changing the temperature, time, and volume of the reaction vehicle (DMF) (Table 2). The successful formation of NS was primarily confirmed by the observation of a gel-like mass during the reaction (gelification) together with the appearance of a deep violet color upon adding ferric chloride [30]. As shown in Table 2, using a small volume of DMF (3 mL) resulted in an incomplete reaction at different reaction conditions (F1–F4). Increasing the DMF volume to 6 mL resulted in the successful formation of NS, except for F5 (90 °C, 2 h). Furthermore, increasing the reaction temperature to 120 and 150 °C allowed for NS formation in the shorter time interval tested (2 h, F7, and F9). Based on the percentage yield (49.85 ± 1.2%), 150 °C was selected as the optimum reaction temperature (F9).

FS loading (FS-NS) was achieved by simple overnight stirring of drug ethanolic solution and the aqueous nano-dispersion of blank NS (at a weight ratio of 1:4) [10].

### 3.2. Preparation of LF-Coated FS-NS

**Figure 1:**
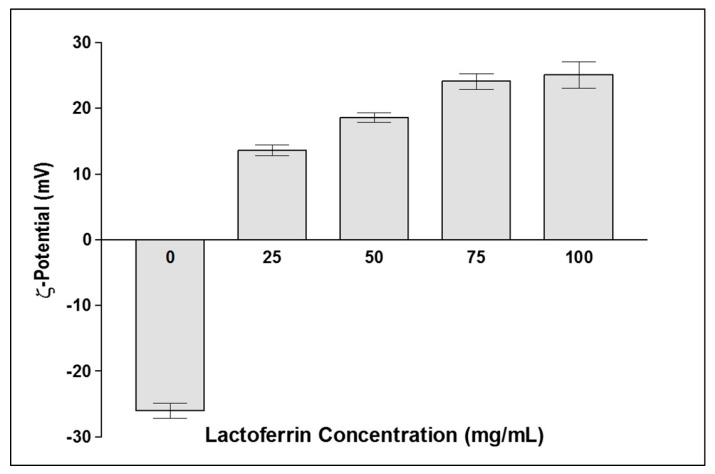
Effect of lactoferrin concentration on FS-NS ζ-potential (mV). Data presented as mean ± SD (n = 3).

LF has been extensively studied as a natural tumor-targeting ligand owing to its ability to bind specific receptors overexpressed on the surface of cancerous cells, in addition to its biocompatibility and biodegradability [29]. In the current work, LF coating on NS was investigated for the first time. This was based on the electrostatic interaction between cationic LF and the negatively charged surface of FS-NS (−26 mV). The effect of the LF concentration added (25–100 mg/mL) on the ζ-potential of FS-NS was studied (Figure 1). Upon increasing the LF concentration from 0 to 75 mg/mL, ζ-potential shifted from −26 ± 6.5 to 24 ± 1.1 mV, indicating the deposition of LF on the surface of NS. Further increases in LF beyond 75 mg/mL only brought about a slight insignificant (*p* ≥ 0.05) change in ζ-potential (25.1 ± 2 mV for 100 mg/mL LF), reflecting complete FS-NS surface coverage with an LF layer. Hence, 75 mg/mL was chosen as the optimum LF concentration for coating FS-NS.

### 3.3. Physicochemical Characterization

#### 3.3.1. Colloidal Properties and Entrapment Efficiency

The colloidal properties of selected NS formulations are shown in Table 3. The optimized blank NS formulation (F9) chosen for further development showed an average PS, PDI, and ζ-potential of 46.1 ± 6.2 nm, 0.13, and −22 ± 0.8 mV, respectively. The negative charge could be attributed to the presence of free β-Cyclodextrin hydroxyl groups and DPC carbonyl groups [10,41]. FS loading into NS resulted in a slight decrease in PS (38.2 ± 3.8 nm) with an insignificant (*p* ≥ 0.05) change in ζ-potential (−26 ± 6.5 mV). These results reflect efficient drug entrapment in the porous NS. LF-coated FS-NS showed a significant increase in PS (*p* ≤ 0.05) compared to the uncoated formulation. The increase in size, together with the shift in ζ-potential from negative to positive, suggests successful coating and deposition of LF on the surface of NS [26]. The high ζ-potential value of the three NS formulations tested allows for sufficient particle repulsion, indicating good colloidal stability [41].

A high entrapment efficiency of FS (>95%) that was unaffected by LF coating, and DL% ~24%, were calculated. (Table 2). High EE% of lipophilic drugs in ß-CD nanosponge formulations have been previously reported for curcumin [42] and quercetin [10]. The high EE% could be ascribed to the porous nature of NS, in which the drug is deposited [43]. 

#### 3.3.2. Analysis of Surface Area and Porosity of NS

**Figure 2:**
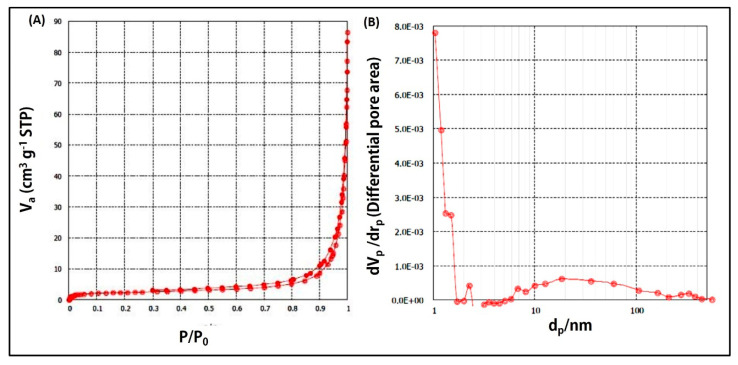
(**A**) Nitrogen adsorption–desorption isotherm; (**B**) pore size distribution of selected NS formulation (F9).

Figure 2 illustrates the nitrogen adsorption–desorption isotherm (Figure 2A) and the pore size distribution (Figure 2B) of the selected NS formulation (F9). A type IV isotherm with a hysteresis loop and an average pore diameter of ~26 nm were obtained, reflecting the mesoporous nature of NS [44]. The total surface area and total pore volume were 15.8 m^2^/g and 0.1 cm^3^/g, respectively. The large surface area and pore volume observed allow for efficient drug loading [31], subsequently supporting the high FS EE% observed.

#### 3.3.3. Microscopical Examination

**Figure 3:**
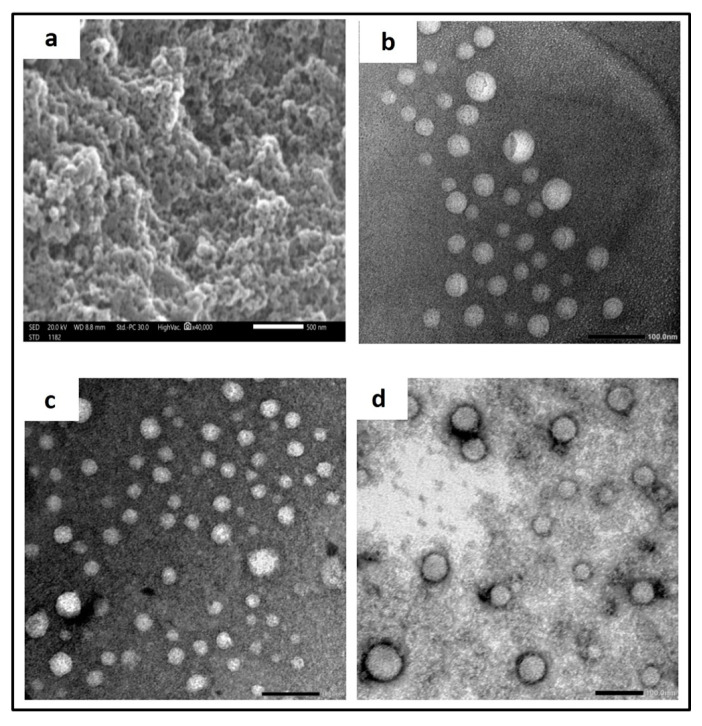
(**a**) SEM of NS with measurement of pore size. Magnification × 40 K, scale bar represents 500 nm. TEM images (**b**–**d**) showing the morphology of (**b**) NS, (**c**) FS-NS, and (**d**) LF-FS-NS. Magnification × 30 K, scale bar represents 100 nm.

SEM imaging showed a rough, porous surface (Figure 3a) with a pore diameter of ~30 nm, confirming the mesoporous structure of NS. TEM micrographs revealed spherical non-aggregated nanoparticles for blank NS (Figure 3b), which was not affected by FS loading except for the decrease in particle size (Figure 3c). A dense coat surrounding the nanosponges could be observed for LF-FS-NS (Figure 3d).

#### 3.3.4. In Vitro Drug Release

**Figure 4:**
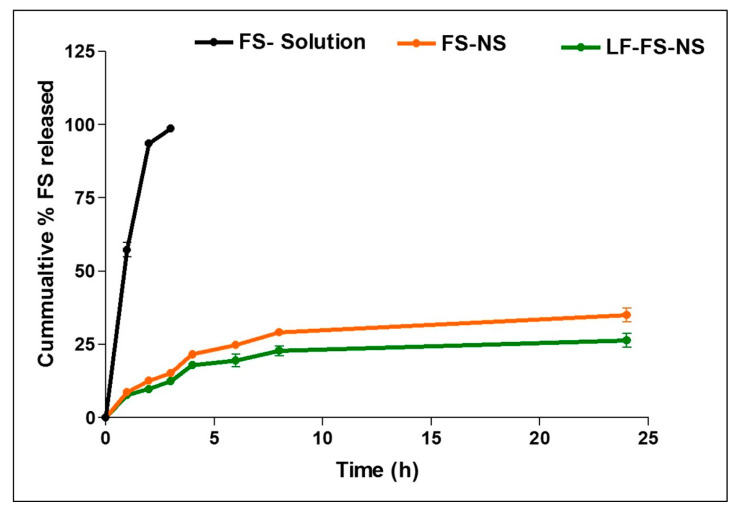
In vitro release profile of FS from FS solution, FS-NS, and LF-FS-NS at 37 °C in PBS (7.4) with 0.1% *w*/*v* Tween^®^ 80. Data represent mean ± SD (n = 3).

In vitro release profiles of FS solution, FS-NS, and LF-FS-NS are shown in Figure 4. FS solution exhibited complete release, reaching 100% after 3 h. On the other hand, FS release from FS-NS and LF-FS-NS was slow and steady, reaching only 35 and 26% after 24 h, respectively. In addition to the prolonged sustained release profile, the low initial burst observed (less than 10%) strongly confirms the presence of FS inside the pores of the nanosponge, as previously stated for other drugs loaded in ß-CD-NS [12,13]. The decrease in the slope of the drug release curve with time reflects the gradual increment of diffusion distance in the polymeric matrix. Moreover, the lower percentage of FS released for LF-FS-NS as compared to FS-NS could be attributed to the additional barrier created by the coating layer restricting release medium diffusion into the NS matrix [45].

The drug release mechanism of FS-NS and LF-FS-NS was determined by fitting to different release kinetics models, namely the zero-order, first-order, Higuchi, Korsmeyer –Peppas, and Hixson–Crowell models. To designate the data best fit, the largest correlation coefficient (r) and smallest mean standard error (MSE) were used as statistical parameters. The results indicated diffusion-controlled FS release from both FS-NS and LF-FS-NS as the greatest r value (0.94), and the minimum MSE value were observed for the Korsmeyer–Peppas model. A release exponent value (n) ≤ 0.5 was observed as being indicative of Fickian diffusion.

#### 3.3.5. Fourier Transform Infrared Spectroscopy (FTIR)

**Figure 5:**
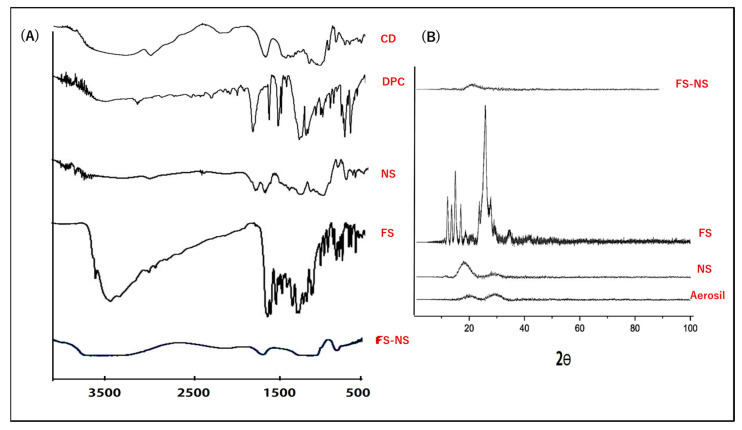
(**A**) FTIR spectra for FS-NS and its components; (**B**) X-ray diffraction pattern of FS, NS, and FS-NS.

FTIR spectra of β-CD, DPC, FS, blank NS, and FS-NS are shown in Figure 5A. β-CD displayed characteristic bands at 3288 for O–H stretching, 2921 for C-H stretching, 1416 for C-H bending, and 1047 cm^−1^ for C–O stretching. The most characteristic band in the FTIR spectrum of DPC is 1771 cm^−1^, corresponding to C=O. In the NS spectrum, the appearance of a new sharp absorption band at 1759 cm^−1^ corresponding to carbonyl (C=O) stretching vibration, which was absent in the FTIR spectrum of pure β-CD, together with the reduction in O–H stretching vibration, indicates carbonate linkage formation with OH groups of β-CD, proving efficient cross-linking with DPC. This is consistent with previous reports [10,12].

The FS spectrum showed absorption bands at 3519 and 3351 cm^−1^ corresponding to aromatic ring attached O–H stretching, 1607 cm^−1^ for C=O stretching, 1571 cm^−1^ for C=C stretching, 1477 cm^−1^ for C–O stretching, and 1279 cm^−1^ for C–O–H bending vibrations [26].

The FTIR spectrum of FS-NS exhibited some changes in the fingerprint region (400–1400 cm^−1^) compared to the FS spectrum. Peak broadening and shifting in the spectrum of FS-NS compared to blank NS suggest definite interactions between FS and β-CD NS, further confirming the effective internalization of the drug into the pores of the formed NS [10]. 

#### 3.3.6. X-ray Diffractometry (XRD) 

To assess drug crystallinity in the developed FS-NS, nondestructive XRD was conducted. Figure 5B shows the XRD patterns of FS, NS, and FS-NS, as well as Aerosil^®^ 200, which was used for drying the aqueous dispersions of tested NS. The Aerosil^®^ 200 diffractogram showed its amorphous structure, as only a broad peak at 22.5° was observed [36]. The FS diffractogram revealed prominent sharp diffraction peaks at 2θ angles 12.5, 15.2, 17.1, 25.7, and 28.5°, reflecting FS’s crystalline nature [46]. These characteristic sharp peaks of FS disappeared upon loading into FS-NS, indicating its presence in an amorphous state which could be attributed to the supramolecular complex formation of FS with NS [47]. 

### 3.4. Cell Line Studies

#### 3.4.1. Cytotoxicity Evaluation

The antiproliferative effect of FS on TNBC has been previously reported [37,48,49]. In this study, the cytotoxicity of FS-NS and LF-FS-NS in comparison with FS solution was evaluated on MDA-MB-231 cells using MTT assay in the concentration range of 10–100 µg/mL. To compare the antitumor activity of FS solution and NS formulations, %viability and, consequently, IC_50_ values were calculated. FS showed concentration-dependent cytotoxicity with an IC_50_ value of 59.18 µg/mL. Interestingly, FS encapsulation into NS (FS-NS) resulted in a 1.3-fold decrease in IC_50_ (45.06 µg/mL). The obtained results are in agreement with previous reports on the promotion of cytotoxicity of different biomolecules following loading into ß-CD-NS formulations [12,13]. Further coating of FS-NS with LF boosted the antiproliferative effect of FS with a reduction in IC_50_ to 27.67 µg/mL (a 2.1- and 1.6-fold decrease compared to FS and FS-NS, respectively). Similar effects of LF modification on cytotoxicity enhancement were shown for PLGA nanoparticles [28], bilosomes [33], and nanostructured lipid carriers (NLC) [50]. This could be partly a result of the innate anticancer effect of LF [29]. Blank formulations (NS and LF-NS) were included in the study and showed percentage MDA-MB-231 cell viability exceeding 80% for all concentrations tested. This proves the cytocompatibility of the blank nano-formulations and provides evidence that the observed cytotoxicity of FS-loaded nanosponge formulations is due to the enhanced cellular interactions of FS following loading into NS [26].

#### 3.4.2. In Vitro Apoptosis Assay

**Figure 6:**
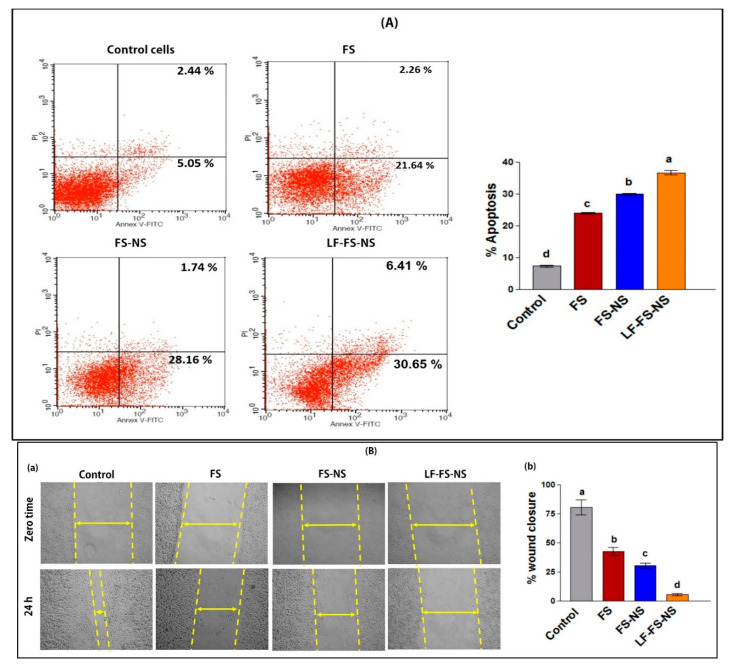
(**A**) Percentage apoptosis for FS, FS-NS, and LF-FS-NS by flow cytometry using annexin V FITC/propidium iodide assay after incubation for 24 h with MDA-MB-231 cells and (**B**) scratch wound assay: (a) migration inhibitory activity of free FS, FS-NS, and LF-FS-NS on MDA-MB-231 cells (magnification ×20) and (b) percentage wound closure. Data were expressed as means ± SD (n = 3). Data were analyzed using one-way ANOVA followed by Tukey’s post-hoc test for group comparisons. Means of similar symbols were statistically insignificant: a > b > c > d (*p* ≤ 0.05).

The apoptosis (programmed cell death)-mediated anticancer effect of FS and FS-loaded nanosponge formulations was investigated using flow cytometry of annexin V-stained apoptotic cells (Figure 6A). FS resulted in a significant (*p* ≤ 0.05) apoptotic activity compared to the control group (23.9 ± 0.17% and 7.3 ± 0.28 %, respectively). Loading into NS formulation brought about a significant increase in FS apoptotic activity (29.9 ± 0.3%) which was further augmented (*p* ≤ 0.05) following LF coating of FS-NS (36.6 ± 0.61%). The significant apoptotic effect of LF-FS-NS compared to the uncoated formulation could somewhat be attributed to the inhibitory effect of LF on plasmalemmal V-H+-ATPase [51]. The higher cellular apoptosis for FS-NS and LF-FS-NS formulations compared to the FS solution elucidates in part the observed enhancement of cytotoxicity.

#### 3.4.3. Cell Migration

FS has been previously shown to suppress migration and metastasis of TNBC through epithelial-to-mesenchymal transition reversal via the PTEN/Akt/GSK3β signaling pathway [20]. In the current study, the ability of FS-NS and LF-FS-NS vs. FS solution to inhibit MDA-MB-321 cell migration was studied by the wound healing assay (Figure 6B). Compared to control cells, FS significantly reduced wound closure by two-fold. This is in agreement with previous reports on the ability of FS to effectively reduce the migration of TNBC cells in the range of 20 to 76% [52]. Interestingly, FS-NS succeeded in further significant (*p* ≤ 0.05) inhibition of wound closure (42.5 ± 3.5 and 30.1 ± 2.1% for FS solution and FS-NS, respectively). LF-FS-NS was the most suppressing formulation, achieving an approximately 15-, 8-, and 6-fold decrease in wound closure compared to the control, FS solution, and FS-NS, respectively.

#### 3.4.4. Cellular Uptake

**Figure 7:**
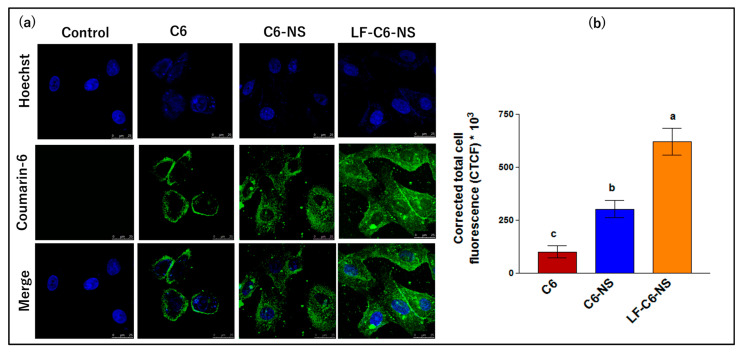
(**a**) Confocal laser scanning microscope images showing cellular uptake of free coumarin 6 solution and its NS formulations after 4 h incubation with MDA-MB-231 cells and (**b**) corrected total fluorescence intensity. Data were expressed as means ± SD (n = 3). Data were analyzed using one-way ANOVA followed by Tukey’s post-hoc test for group comparisons. Means of similar symbols were statistically insignificant: a > b > c (*p* ≤ 0.05).

The extent of the internalization of C6-labeled NS and LF-NS by MDA-MB-231 cells was evaluated by confocal microscopy (Figure 7). Following 4 h exposure, the free dye was internalized by the cells; however, C6 loading in NS resulted in a 3- and 6-fold increase in cellular uptake for the uncoated and LF-coated formulations, respectively. The improvement in cellular uptake following FS loading into NS and LF-NS could partially explain the enhanced antiproliferative, apoptotic, and migration-inhibitory effects observed. Enhanced cellular uptake of the nano-formulations could be attributed to their small size (below 100 nm) which allows for endocytosis and not just simple diffusion as previously reported [33]. The superior cellular uptake observed for LF-C6-NS compared to C6-NS reflects faster and more efficient internalization of the formulation into breast cancer cells due to LF interaction with its target receptors overexpressed on metabolically active cancer cells [28]. Moreover, LF’s positive charge allows for cell entry via electrostatic interaction with the negatively charged cell membrane glycosaminoglycans [53]. Surface charge was shown to not only affect cellular uptake rates but also intracellular trafficking [50].

### 3.5. In Vivo Studies

#### 3.5.1. Pharmacokinetic Study

**Figure 8:**
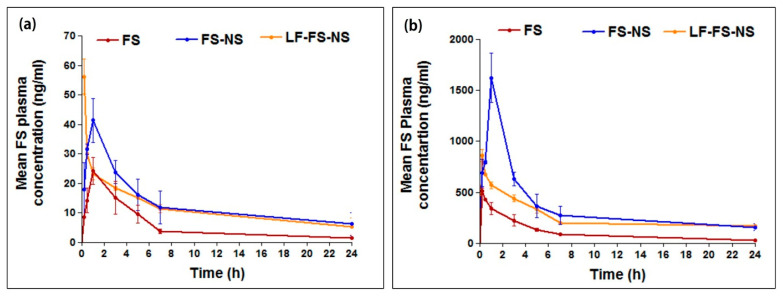
Mean FS plasma concentration–time profiles of FS solution and NS formulations following (**a**) oral and (**b**) intraperitoneal administration of a single dose (35 mg/kg) to rats. Data represent mean ± SD (n = 6).

The bioavailability of FS administered to rats either orally or by IP injection was studied for free FS, FS-NS, and LF-FS-NS (Table 4, Figure 8). The drug plasma concentration–time profiles after oral administration of a single FS dose (30 mg/kg) either free or loaded into NS formulations are demonstrated in Figure 8a, and the calculated pharmacokinetic parameters are listed in Table 4. Results revealed marked changes in the pharmacokinetic behavior of FS after loading into both uncoated and LF-coated NS. A significant decrease in T_max_ was observed for LF-FS-NS (0.25 h) compared to FS suspension and FS-NS (1 h) indicating a faster rate of absorption. This might be attributed to the positive charge on LF-FS-NS, which allows for favorable distribution in the small intestine and uptake via multiple endocytosis pathways compared to negatively charged nanoparticles [54]. Furthermore, positively charged nanoparticles were shown to electrokinetically interact with mucus, thus, opening epithelial cells’ tight junctions and promoting absorption via the paracellular pathway [55].

Whereas FS loading into uncoated NS did not affect T_max_, a significant increase in C_max_ by 1.7-fold (41.3 ± 7.4 ng/mL) compared to FS suspension (24.3 ± 4.6 ng/mL) (*p* ≤ 0.001) was observed. Moreover, LF-FS-NS resulted in a 2.3-fold increase in C_max_ (56.2 ± 5.9 ng/mL) compared to FS suspension, which was also significantly higher than the uncoated formulation, (*p* ≤ 0.01). Again, both NS formulations achieved a significant increase in AUC _0–∞_, with 3.1- and 2.5-fold for the uncoated and LF-coated NS, respectively, compared to drug suspension (*p* ≤ 0.05), with a significant increase in t_1/2_ (*p* ≤ 0.05). It is noteworthy that LF-FS-NS exhibited a significantly lower t_1/2_ compared to FS-NS (*p* ≤ 0.05), owing to its cationic nature, which could facilitate serum protein aggregation, opsonization, and, thus, systemic clearance by macrophages [56]. Despite their difference in t_1/2_, the differences between AUC for FS-NS and LF-FS-NS did not reach statistical significance.

The pharmacokinetic behavior of FS-NS and LF-FS-NS vs. FS suspension was also investigated following IP administration. Figure 8b shows the drug plasma concentration–time profiles and Table 4 demonstrates the main pharmacokinetic parameters. Following IP administration, an obvious increase in the amount and extent of FS reaching the circulation and, hence, bioavailability, was observed for NS formulations compared to FS suspension, as indicated by the significantly higher C_max_ (3-fold for FS-NS and 1.7-fold for LF-FS-NS) and AUC_0–∞_ (4.3-fold for FS-NS and 3.2-fold for LF-FS-NS) (*p* ≤ 0.05). Furthermore, FS half-life was significantly prolonged following its loading into either coated or uncoated NS formulations, (*p* ≤ 0.05). Nevertheless, t_1/2_ for FS-NS was significantly higher than that of LF-FS-NS (*p* ≤ 0.001) in a similar manner to that observed following its oral administration, suggesting a possible systemic clearance by macrophages being positively charged [56]. 

The improved drug bioavailability following administration of FS-NS formulations via either oral or IP routes compared to FS suspension matches previous reports on the potential of NS to enhance erlotinib bioavailability as a result of supramolecular complex formation between the drug and porous NS [57]. Drug inclusion within the nanocavities reduced its particle size and, accordingly, increased solubility and dissolution rate, hence, facilitating absorption [57]. Furthermore, being deeply incorporated in the nanopores could possibly allow for the avoidance of drug pre-systemic intestinal and first-pass hepatic metabolism [57]. 

It is worth mentioning that FS bioavailability was significantly higher via the IP route compared to the oral one when administered either as a suspension or loaded into NS formulations. This could be attributed to by-passing the drug pre-systemic intestinal metabolism following IP injection [58]. Furthermore, IP administration allows for a higher and faster drug absorption rate due to the rapid uptake of drug from the peritoneal cavity, resulting in a more rapid saturation of the drug-metabolizing enzymes than following oral administration. Consequently, higher concentrations of the unmetabolized drug will be available in systemic circulation, resulting in higher drug bioavailability [59]. 

#### 3.5.2. In Vivo Evaluation of Anticancer Potential

The anticancer efficacy of different NS formulations was assessed on female mice bearing Ehrlich ascites tumors, a well-established model of spontaneous murine mammary adenocarcinoma [60]. Efficacy assessment started when tumor size reached ~100 mm^3^. Treatment was administered by IP injection over 14 days.

##### Tumor Growth Inhibition

**Figure 9:**
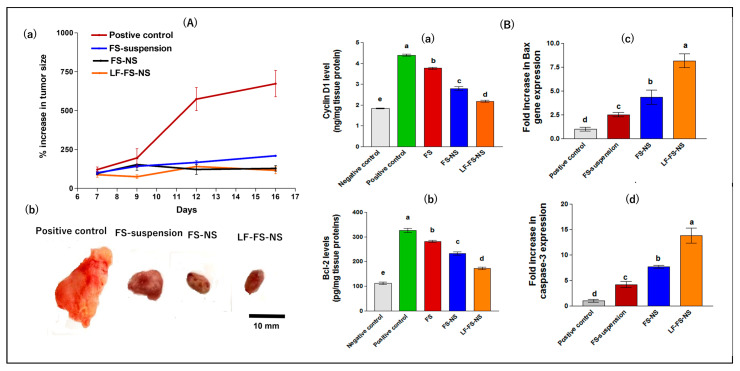
(**A**) Antitumor effect of FS and NS formulations in comparison with untreated control 16 days post-IP treatment of Ehrlich ascites mammary tumor in mice showing: (**a**) percentage change in tumor size relative to pretreatment volume and (**b**) digital images of excised tumors. (**B**) Tumor biomarker levels following 16-day treatment with FS and FS-NS formulations compared to untreated control: (**a**) cyclin D1, (**b**) Bcl-2, (**c**) Bax, and (**d**) caspase-3. Data were expressed as means ± SD (n = 3). Data were analyzed using one-way ANOVA followed by post-hoc Tukey’s test for group comparisons. Means of similar symbols were statistically insignificant: a > b > c > d > e (*p* ≤ 0.05).

Figure 9A(a) demonstrates the percentage increase in tumor size for different study groups during the study period. Representative photographs of excised tumors following sacrifice are shown in Figure 9A(b). The positive control mice exhibited a significant increase in tumor size throughout the study period, with ~600% after 16 days (*p* ≤ 0.01). Tumor growth inhibitory effect was achieved following treatment with FS suspension, FS-NS, and LF-FS-NS, with a percentage increase in tumor size of 209.4, 128.9, and 116.7 %, respectively, after 16 days, which is consistent with the excised tumor images (Figure 9A(b)).

##### Assessment of Tumor Biomarkers

Cyclins are a family of cell proteins controlling cell cycle progression and promoting tumor proliferation with cyclin D1 (CD1) being a cell-cycle regulator essential for G1-phase progression. It is overexpressed in more than 50% of breast tumors [61]. In the current study, the positive control group demonstrated a significant overexpression in CD1 level by 2.4-fold compared to the negative control group (*p* ≤ 0.001) (Figure 9B(a)). Conversely, all treated groups showed a significant reduction in CD1 level compared to the positive control (*p* ≤ 0.001) in the following order: FS suspension < FS-NS < LF-FS-NS. The FS effect on lowering CD1 transcription in breast cancer is consistent with previous reports [26]. This effect was significantly augmented for FS-NS, which could be explained by the FS bioavailability enhancing effect of NS and the improved cell penetration via the EPR effect [62,63]. The LF-FS-NS formulation achieved the lowest expression of CD1, confirming its superior cytotoxic potential.

Intrinsic apoptosis, or programmed cell death, includes initial mitochondrial perturbation arising from cytotoxicity and is mainly regulated by the Bcl-2 protein family [64]. The latter is subdivided into pro-apoptotic proteins, such as Bax, and anti-apoptotic ones, such as Bcl-2. The affinities and relative abundance of different Bcl-2 proteins control whether anti-apoptotic or pro-apoptotic reactions predominate [64]. Overexpression of Bcl-2, present mainly on the outer membrane of the mitochondria, protects against apoptosis induced by many cytotoxic agents [65]. On the contrary, the increased expression of the pro-apoptotic proteins, such as Bax, will stimulate the release of mitochondrial cytochrome C into the cytoplasm and, subsequently, the activation of caspase-3 [65]. Caspase-3, a key mediator of apoptosis, is a frequently activated death protease that catalyzes the specific cleavage of several vital cellular proteins [66]. In this context, the levels of Bcl-2, Bax, and caspase-3 biomarkers were quantitively determined in tumor tissues to investigate the possible molecular pathway for the anticancer activity of FS. As shown in Figure 9B(b,c), the positive control group showed a significant up-regulation in the levels of the anti-apoptotic protein Bcl-2 by ~3-fold compared to the negative control group (*p* ≤ 0.001). Contrarily, FS suspension significantly decreased the levels of Bcl-2 by 13.5% and increased the expression of the Bax gene by ~2.5-fold compared to the positive control group (*p* ≤ 0.05), which is in agreement with previously reported data [22,49]. Furthermore, FS loading into NS resulted in a significant down-regulation in Bcl-2 levels by 17.6% with an up-regulation in Bax gene expression by 1.7-fold compared to FS suspension (*p* ≤ 0.05), elucidating the role of supramolecular complex formation between FS and NS in enhancing FS anticancer activity. Interestingly, LF-FS-NS significantly achieved a higher reduction in Bcl-2 levels by 26.2% and an increase in Bax gene expression (1.9-fold) vs. uncoated FS-NS (*p* ≤ 0.05). 

To further confirm the apoptotic capability of different treatment groups, analysis of the caspase-3 gene was carried out. As previously reported, FS-induced apoptosis acts through mitochondrial- and caspase-3-dependent pathways [67]. As shown in Figure 9B(d), all treatments significantly increased the expression of the caspase-3 gene with variable degrees vs. the positive control group (*p* ≤ 0.05). This could be due to the proven ability of FS to up-regulate Bax gene expression and, subsequently, the induction of apoptosis via caspase-3 activation [67]. Again, the LF-coated formulation exhibited the highest elevation in caspase-3 expression, reflecting superior efficacy. This could be explained by its ability to actively target LF receptors overexpressed on breast cancer cells [28], thus, allowing for efficient FS cellular uptake and internalization, as demonstrated by the cellular uptake study (Section 3.4.4). Moreover, on the subcellular level, LF can increase drug nuclear localization, hence, achieving optimum efficacy, as the nucleus is the main site of action for most anticancer drugs [53]. This highlights the potential of NS surface modification with LF to improve the anticancer activity of FS via an active targeting mechanism. Indeed, LF-targeted formulations have previously shown higher anticancer activity compared to both free drug and untargeted nanotherapy [33,56].

##### Histopathological Evaluation

**Figure 10:**
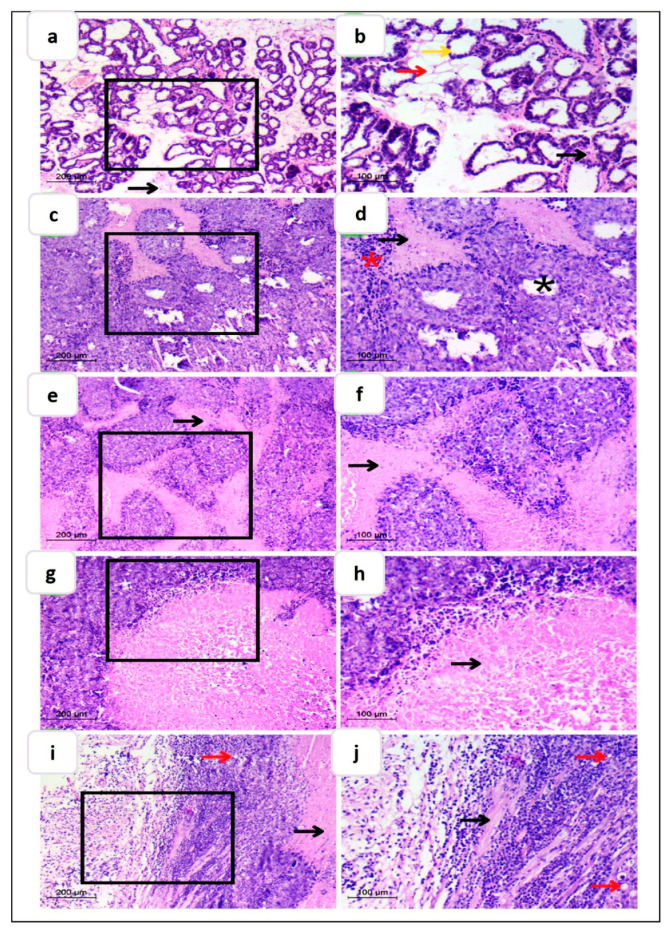
Photomicrographs illustrating H&E-stained breast tissue specimens of (**a**,**b**) normal breast and (**c**,**d**) positive control group and groups treated with (**e**,**f**) FS suspension, (**g**,**h**) FS-NS, and (**i**,**j**) LF-FS-NS. Yellow arrow: ductal system. Red arrow: adipose connective tissue. Black arrow: connective tissue stroma. Black star: degeneration. Red star: lymphocytes. The right panel represents a higher magnification of the area selected in the left panel. Magnification ×100, scale bar 200 µm (low)- ×200, scale bar 100 µm (high).

Findings of the tumor growth inhibition study and tumor biomarkers were additionally verified via histopathological examination of excised tumor tissue (Figure 10). Examined sections from normal control groups (Figure 10a,b) showed normal breast tissue architecture. Glands were organized into lobules of complex branching alveolar glands with extensive interlobular connective tissue and fat between them. The stromal compartment predominated and is packed with adipocytes, which offered insulation and aided in the protection of the fragile mammary gland tissue. The ductal lobular system’s cellular lining was bilayered and composed of inner (luminal) epithelial cells, which were cuboidal to columnar in shape and had a pale eosinophilic cytoplasm. Outer (basal) myoepithelium cells were variable in form, ranging from flattened cells with compressed nuclei to prominent epithelioid cells with a copious transparent cytoplasm, and could occasionally have a myoid appearance. 

Conversely, the typical lobular and ductal architecture was lost in the positive control group (Figure 10c,d), which revealed the highest intraductal proliferation in the gland, as evidenced by the formation of irregular dark proliferation sites with aberrant nuclei and a mild appearance of lymphoid tissue. Moreover, there was little evidence of fibrous interductal stroma, indicating medullary cancer. Furthermore, myoepithelium loss was seen as an indication of invasion.

Treatment with FS suspension influenced the histopathological characteristics by the formation of some intralobular connective tissue but still condensed in a non-fibrous aspect with obvious proliferating ductal glands showing lumen degradation, reflecting minor efficacy (Figure 10e,f). On the other hand, treatment with different FS-loaded NS formulations presented variable effectiveness. In this respect, FS-NS accomplished lower efficacy, as indicated by the decrease in the number of proliferative cells, but aberrant stroma and many lymphocytes were still seen (Figure 10g,h). Interestingly, LF-FS-NS demonstrated the greatest anticancer potential, as indicated by the absence of any proliferative cells, but still showed disorganized cells (Figure 10i,j) owing to the short treatment time. Some adipose tissue started to be retrieved. Nearly normal breast tissue architecture was seen. Additionally, the interlobular stroma appeared normal with many lymphocytes.

To sum up, combined results of biochemical and histopathological evaluation verified the superiority of LF-FS-NS in the treatment of breast cancer compared to both FS suspension and uncoated formulation, suggesting successful tumor targeting and drug accumulation in cancer cells.

#### 3.5.3. In Vivo Toxicity 

All mice treated with FS test formulations survived and appeared healthy throughout the study. An insignificant change in body weight (*p* ≥ 0.05) was observed. Evaluation of liver and kidney functions was performed, and the results are described in Table 5. Liver enzymes, namely ALT and AST, were assessed as indicators for proper liver performance, while serum levels of both urea and creatinine were measured as indicators of renal function. Results demonstrated insignificant changes in the measured parameters for all tested groups compared to normal healthy control mice (*p* ≥ 0.05), suggesting the safety of the developed formulations. Our results were in accordance with many reported articles describing the safety and biocompatibility of ß-CD-NS [68,69].

**Figure 11:**
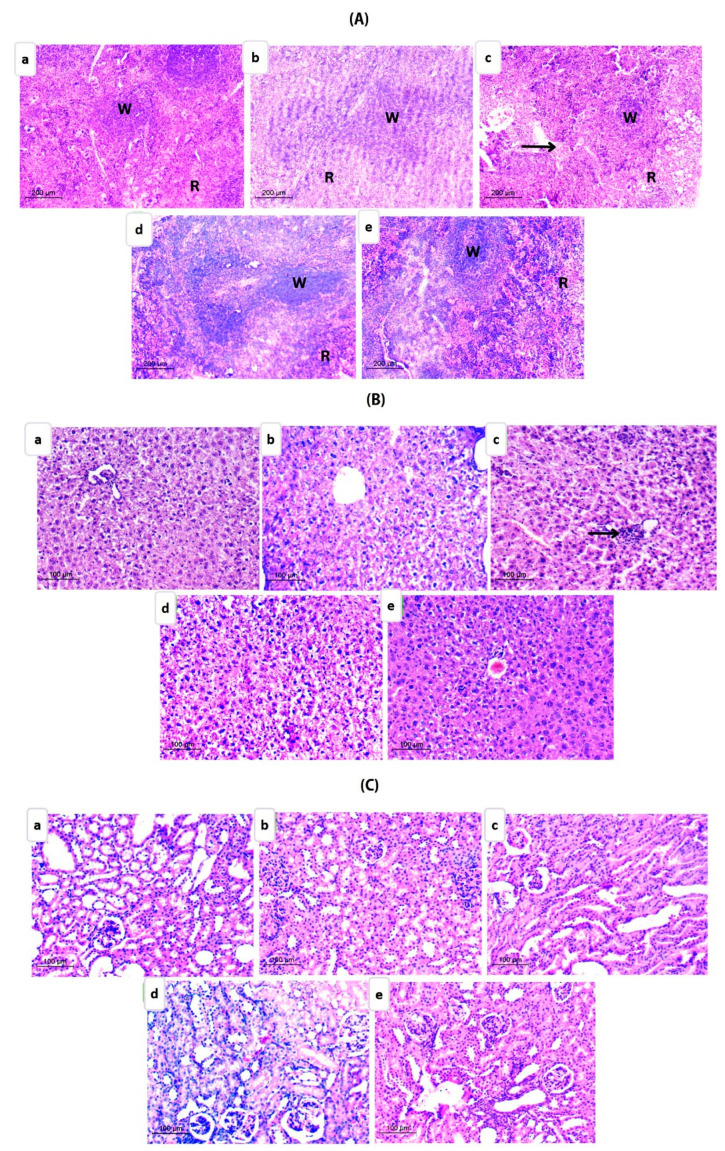
Photomicrographs illustrating H&E-stained (**A**) spleen tissue specimens of (**a**) normal group, (**b**) positive control group, (**c**) FS suspension-treated group, (**d**) FS-NS-treated group, and (**e**) LF-FS-NS-treated group. Black arrow: hemorrhage. W: white pulp. R: red Pulp. (**B**) Liver tissue specimens of (**a**) normal group, (**b**) positive control group, (**c**) FS suspension-treated group, (**d**) FS-NS-treated group, and (**e**) LF-FS-NS-treated group. Black arrow: inflammation. (**C**) Kidney tissue specimens of (**a**) normal group, (**b**) positive control group, (**c**) FS suspension-treated group, (**d**) FS-NS-treated group, and (**e**) LF-FS-NS-treated group. Magnification ×100 spleen-×200 liver and kidney.

To further assess the in vivo toxicity, histopathological examination of the most affected organs (liver, kidney, and spleen) was performed at the end of treatment (Figure 11A–C). The spleen sections (Figure 11A) isolated from different study groups showed almost normal architecture, with a well-delineated white and red pulp with continuous trabecular throughout the tissues encountered by a capsule. The white pulp included lymphoid follicles as well. Nonetheless, the FS suspension group showed some dilatation and the development of bleeding (Figure 11A(c)).

Liver sections for FS-loaded NS groups demonstrated typical hepatocyte appearance with normal cells in the center having polyhedral shape, vacuolated acidophilic cytoplasm, and rounded vesicular nuclei (Figure 11B(d,e)). However, examined specimens from the FS suspension group exhibited mild inflammation near the central vein (Figure 11B(c)).

Despite the extensive reports on the nephroprotective role of FS [70], the renal tissue was the most affected by FS suspension (Figure 11C(c)). Since this was only obvious based on histopathological but not biochemical analysis, the slight changes observed could be attributed to different factors, including mice physiological state and other effects on nutrients uptake, among others [68]. Interestingly, renal tissues showed no symptoms of toxicity for FS-NS formulations (Figure 11C(d,e)). Normal appearance of (Malpighian) corpuscles was evident, which were comprised of glomerular capillaries and Bowman’s capsules with subcapsular space. Many proximal convoluted tubules were lined with simple truncated cubical (pyramidal) cells with basal spherical nuclei and had narrow lumina. The lumina in distal convoluted tubules were large.

## 4. Conclusions

In the current study, ß-CD nanosponges cross-linked with diphenyl carbonate were selected for improving the bioavailability and anticancer potential of fisetin. For the first time, to the best of our knowledge, FS-NS was coated with the active targeting ligand LF (LF-FS-NS). The optimized formulations of FS-NS and LF-FS-NS were shown to enhance FS bioavailability both orally and via IP injection. NS formulations administered by IP significantly enhanced the efficacy of FS against breast cancer. This was verified at both the cellular level on MDA-MB-231 cells and the molecular level in an Ehrlich ascites tumor compared to FS suspension, with superior effects achieved by the actively targeted formulation (LF-FS-NS). Accordingly, LF-FS-NS offers a highly adaptable strategy for bioactive targeted nanotherapy for the delivery of FS and possibly other phytomedicines to treat breast cancer.

## Figures and Tables

**Table 1:** qRT-PCR primers used to evaluate gene expression levels of Bax and caspase-3 in tumor tissues.

Gene	Forward	Reverse
Bax	GCTGACATGTTTGCTGATGG	GATCAGCTCGGGCACTTTAG
Caspase-3	AGGGGTCATTTATGGGACA	TACACGGGATCTGTTTCTTTG
GAPDH	TCACCACCATGGAGAAGGC	GCTAAGCAGTTGGTGGTGCA

**Table 2:** Optimization of cross-linking reaction conditions.

Formulation Code	DMF Volume (mL)	Reaction Temperature (°C)	Reaction Time (h)	Ferric Chloride Test	Gelification
F1	3	90	2	Negative	No
F2	3	90	5	Negative	No
F3	3	120	2	Negative	No
F4	3	120	5	Negative	No
F5	6	90	2	Negative	No
F6	6	90	5	Positive	Yes
F7	6	120	2	Positive	Yes
F8	6	120	5	Positive	Yes
F9	6	150	2	Positive	Yes
F10	6	150	5	Positive	Yes

**Table 3:** Physicochemical properties of the selected nanosponge formulations (n = 3).

Formulation	Size (nm)	PDI	ζ-Potential (mV)	EE%	DL%
**NS**	46.1 ± 6.2	0.13	−22 ± 1.8	-	-
**FS-NS**	38.2 ± 3.8	0.19	−26 ± 6.5	96.1 ± 0.3	23.8 ± 0.2
**LF-FS-NS**	52.7 ± 7.2	0.09	24 ± 1.1	95.9 ± 0.2	-

**Table 4:** Pharmacokinetic parameters of FS after oral and IP administration of a single dose (30 mg/kg) of FS suspension and NS formulations in rats.

	Oral			Intraperitoneal		
Parameter	FS Suspension	FS-NS	LF-FS-NS	FS Suspension	FS-NS	LF-FS-NS
**t_1/2_ (h)**	7.2 ^c^ ± 1.5	15.4 ^a^ ± 3.5	11.2 ^b^± 2.1	8.5 ^c^ ± 2.1	17.7 ^a^ ± 2.5	12.2 ^b^ ± 1.2
**T_max_ (h)**	1	1	0.25	0.25	1	0.25
**C_max_** **(ng/mL)**	24.3 ^c^ ± 4.6	41.3 ^b^ ± 7.4	56.2 ^a^ ± 5.9	509.8 ^c^ ± 31.6	1622.4 ^a^ ± 242.9	862.9 ^b^ ± 60.1
**AUC _0–24_** **(ng/mL × h)**	135.1 ^b^ ± 4.2	312.1^a^ ± 116.8	274.1 ^a^ ± 18.5	2538.8 ^b^ ± 121.6	8474.5 ^a^ ± 1834.9	6160.1 ^a^ ± 121.9
**AUC _0–∞_** **(ng/mL x h)**	149.5 ^b^ ± 2.7	457.6 ^a^ ± 251.5	368.7 ^a^ ± 28.5	2874.6 ^c^ ± 254.1	12,371.5 ^a^ ± 2143.5	9126.1 ^b^ ± 565.1
* Relative bioavailability		3.1	2.5		4.3	3.2

The study was conducted on female Wistar rats with six animals in each group. Values were expressed as mean ± SD. Data were analyzed using one-way (ANOVA) followed by post-hoc test (Tukey’s). The level of significance was set at *p* ≤ 0.05. Means of similar symbols are statistically insignificant, a > b > c. * Relative bioavailability was calculated by dividing AUC _0–∞_ of different NS formulations to that of FS suspension.

**Table 5:** Effect of free FS suspension and NS formulations on liver and kidney function tests.

Group	ALT (U/mL)	AST (U/mL)	Urea (mg/dL)	Creatinine (mg/dL)
**Negative control**	85.5 ± 2.9	60.1 ± 5.5	43 ± 2.4	0.94 ± 0.1
**Positive control**	92.1 ± 5.1	60.5 ± 2.8	48 ± 3.8	1 ± 0.3
**FS suspension**	91.5 ± 4.3	61.6 ± 5.2	44 ± 3.5	1.07 ± 0.2
**FS-NS**	90.3 ± 6.2	62.2 ± 3.6	49.2 ± 4.6	1.05 ± 0.2
**LF-FS-NS**	91.8 ± 3.4	61.2 ± 6.1	47 ± 2.2	1.2 ± 0.2

The study was conducted on female albino mice with six animals in each group (n = 6). Values were expressed as mean ± SD. Data were analyzed using one-way ANOVA followed by a post-hoc test (Tukey’s) for group comparisons.

## Data Availability

The data presented in this study are available in the article.
